# Great strides, yet a long way to go: a comparative analysis of WASH conditions and associated sociodemographic factors from national hygiene surveys, 2014 and 2018

**DOI:** 10.1080/16549716.2025.2611646

**Published:** 2026-02-02

**Authors:** Abul Kasham Shoab, Mizanul Islam Nasim, Titly Sen, Farjana Jahan, Mahbub-Ul Alam, Supta Sarker, Jesmin Sultana, Rizwana Khan, Khairul Islam, Hasin Jahan, Golam Rasul, Mahbubur Rahman

**Affiliations:** a Environmental Health and WASH, Health System and Population Studies Division, International Centre for Diarrhoeal Diseases Research, Bangladesh (ICDDR,B), Dhaka, Bangladesh; b School of Civil Engineering, University of Leeds, Leeds, UK; c Infectious Disease Division, International Centre for Diarrheal Diseases Research, Bangladesh (ICDDR,B), Dhaka, Bangladesh; d WaterAid, Bangladesh; e Global Health and Migration Unit, Department of Women’s and Children’s Health, Uppsala University, Uppsala, Sweden

**Keywords:** drinking water access, sanitation facilities, hygiene practices, sociodemographic determinants, WASH policy implications

## Abstract

**Background:**

Bangladesh faces substantial inequalities in water, sanitation, and hygiene (WASH), with disparities across sociodemographic groups and between urban and rural populations. Evidence on temporal changes in household WASH access and its determinants remains limited.

**Objective:**

To assess changes in household WASH and examine the influence of sociodemographic factors on access, using data from two national hygiene surveys at national and urban–rural levels.

**Methods:**

In this repeated cross-sectional study, differences in WASH outcomes between the 2014 National Hygiene Baseline Survey and the 2018 National Hygiene Survey were assessed using prevalence differences (PD), and associations with sociodemographic factors were examined using generalized estimating equations (GEE).

**Results:**

From 2014 to 2018, rural households maintained near-universal basic drinking water, while urban households showed a slight decline. Basic sanitation increased substantially in rural areas (PD = 27.8), driving national gains (PD = 25); urban changes were nonsignificant. Basic hygiene improved minimally across all levels. Higher socio-economic status was linked to better WASH outcomes, while larger households had poorer status. Rental housing was associated with unimproved drinking water (Coef.: 1.9) and lower basic sanitation (Coef.: −0.9) but better overall hygiene than self-owned homes. Urban households had lower access to basic drinking water and sanitation, yet better basic hygiene facilities than rural households.

**Conclusion:**

Household WASH improved substantially, especially in rural sanitation and hygiene, while urban areas showed stagnation. Socio-economic status, household size, and housing tenure are key determinants, highlighting the need for targeted interventions to ensure equitable, universal WASH coverage.

## Paper Context

- **Main findings:** Rural areas saw significant improvements in sanitation and hygiene from 2014 to 2018, while urban areas faced stagnation or declines; sociodemographic factors like wealth, household size, and rental status influenced WASH access.
- **Added knowledge:** The study enhances our understanding of how sociodemographic factors, like wealth and housing tenure, impact WASH access, with a focus on addressing urban and rental household inequalities going forward.
- **Global health impact for policy and action:** The findings highlight the need for targeted policies to address WASH disparities, particularly in urban areas and among disadvantaged groups, to achieve universal coverage and meet global health targets.

## Background

Safe water, sanitation, and hygiene (WASH) are essential for health, especially for vulnerable populations. Access to safe water, sanitation, and good hygiene practices not only reduces the burden of communicable diseases but also promotes social and economic development and enhances the overall quality of life [1–3]. Despite progress, global WASH inequalities remain substantial. In 2024, about 1.4 billion people relied on basic drinking water services, 287 million had limited access, 302 million used unimproved sources, and 106 million depended on surface water. Regarding sanitation, 1.9 billion had basic services, 560 million had limited access, 555 million used unimproved facilities, and 354 million practiced open defecation. Around 1.7 billion people worldwide also lacked basic hygiene facilities, with 1 billion having limited services and 611 million having no facility [4].

Unsafe WASH contributes significantly to morbidity and mortality. An estimated 1.4 million deaths (2.5% of global mortalities) and 74 million disability-adjusted life-years (DALYs) were attributable to unsafe WASH in 2019. These burdens are linked to diseases, such as diarrheal diseases, respiratory infections, undernutrition, and helminthiases, primarily affecting low- and middle-income countries (LMICs) [5]. These figures underscore the urgent need for accelerated progress toward achieving Sustainable Development Goal (SDG) 6, which aims to ensure the availability and sustainable management of water and sanitation for all by 2030 [4,6].

As of 2024, an estimated 38% of the population in low-income countries lacked access to basic drinking water, 65% lacked basic sanitation, and 66% lacked basic hygiene services. In low- and middle-income countries (LMICs), the corresponding figures were 9%, 25%, and 23%, respectively. Significant urban–rural disparities persist across these settings, with rural populations generally having lower levels of WASH access [4,7,8]. A study in South Asia found that urban households have higher access to WASH services than rural ones, with wealthier households and household characteristics, such as age, sex of the head, and family size further influencing access [9]. Similar patterns have been reported in developing countries worldwide [7,8,10].

Bangladesh, a South Asian low- and middle-income country (LMIC), must make substantial progress to ensure safe and equitable WASH accesses for its population. Data from the Bangladesh Multiple Indicator Cluster Survey (MICS) 2019 show pronounced inequalities in basic WASH services, with the poorest households having 4.4 times less access than the richest quintile [11,12]. According to the 2024 WHO–UNICEF Joint Monitoring Programme (JMP) report, nearly the entire population had access to at least basic drinking water, while basic sanitation coverage reached 68% in both rural and urban areas. Access to basic hygiene facilities was 66% in rural areas and 80% in urban areas [4]. Disparities in WASH access by demographic, socioeconomic, and regional factors remain evident [9,13]. A recent study found that households in the wealthiest quintile, those in rural areas, and those with five or more members were more likely to have basic WASH facilities [13]. Bangladesh continues to face multiple WASH challenges, including the impacts of climate change, rapid urbanization, aging and insufficient infrastructure, technical and financial inefficiencies, and a fragmented research landscape [14–16].

To address these issues, the government has implemented various policies, such as the Sector Development Plan (FY 2011–25), introduced in 2011, the National Strategy for Water Supply and Sanitation 2014, and the National Strategy for Water Supply and Sanitation 2021 [14,17–19]. To assess WASH access scenario across the country, Bangladesh conducted its first national hygiene survey in 2014. The survey was led by the International Centre for Diarrhoeal Disease Research, Bangladesh (icddr,b), with support from WaterAid Bangladesh and the Policy Support Unit (PSU) of the Local Government Division, Ministry of Local Government, Rural Development, and Cooperatives [20]. Four years later, a second national hygiene survey was conducted in 2018 by the Bangladesh Bureau of Statistics (BBS), with assistance from WaterAid Bangladesh and UNICEF Bangladesh [21].

Despite notable national progress, evidence on temporal changes in household WASH access, the influence of sociodemographic factors, and urban–rural disparities in Bangladesh remains limited. No previous study has combined data from both national hygiene surveys to examine these trends. Addressing this gap is essential for evaluating policy effectiveness, guiding targeted interventions, and advancing progress toward universal WASH coverage. Therefore, the objective of this study is to assess changes in household WASH status in Bangladesh between 2014 and 2018 and to examine the influence of sociodemographic factors on access, using data from the two national hygiene surveys at both the national level and disaggregated by urban and rural settings. The findings are intended to inform evidence-based policymaking, enhance interventions for vulnerable populations, and support national and global efforts toward equitable WASH access and the achievement of Sustainable Development Goal 6.

## Methods

### Data sources

**Figure 1.**
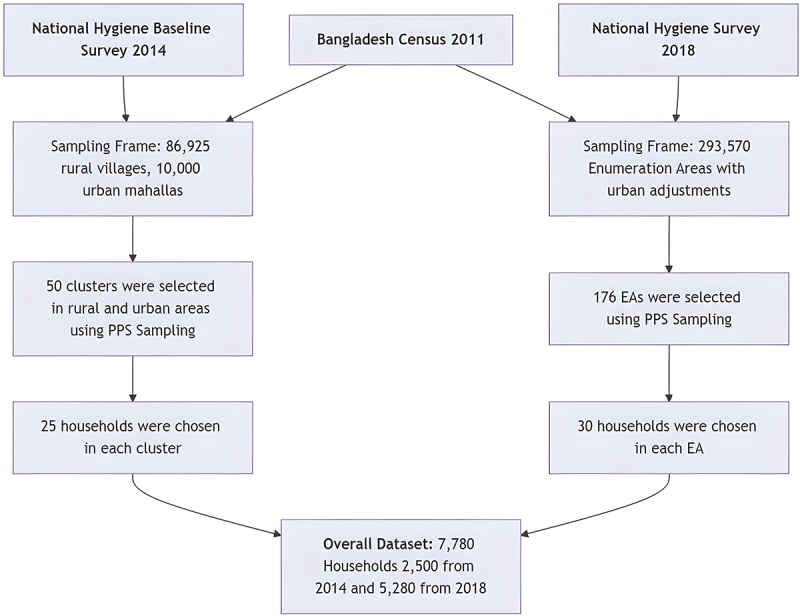
Process of merging National Hygiene Baseline Survey 2014 and the National Hygiene Survey 2018 datasets.

Data sources for this repeated cross-sectional study were the National Hygiene Baseline Survey 2014 and the National Hygiene Survey 2018 [20,21]. Both surveys comprised four data collection categories: household-level hygiene, school hygiene including menstrual hygiene management (MHM) [22,23], food hygiene in restaurants and among street food vendors [24], and hygiene at health facilities [25]. This study investigated WASH status among rural and urban households in both surveys, which collectively provided nationally representative findings for those respective periods. Appending all samples (2500 from the 2014 survey and 5280 from the 2018 survey, for a total of 7780 households) from both surveys created the dataset for the secondary analysis of this study. Figure 1 illustrates the process of merging the two survey datasets.

### 2014 baseline survey methods

Using an assumed design effect of 12.0, 80% power, and a significance level of 0.05, the 2014 survey team calculated the sample size for both rural and urban households. Previous surveys indicated that the prevalence of the handwashing indicator, defined as the availability of soap or ash and water at a convenient handwashing location after defecation, was 47% in rural areas and 44% in urban areas. With a minimum detectable difference of 6%, the required sample size was estimated at 2400 households for 2014 survey; ultimately, 2500 households were surveyed, evenly divided between rural and urban areas [20].

A two-stage stratified cluster sampling design was employed, with Bangladesh divided into rural and urban strata. The sampling frame included 86,925 rural villages and 10,000 urban mahallas (the smallest urban units, comparable to villages). From each stratum, 50 clusters were randomly selected using probability proportional to size (PPS) sampling. In urban areas, clusters corresponded to mahallas, and the sampling followed the methodology of the 2006 Urban Health Survey. Similarly, 50 rural villages were selected based on data from the 2011 Population and Housing Census. Within each selected cluster, 25 households with children under five were chosen, with caregivers serving as the primary respondents. Data collection, conducted from March to June 2014, involved face-to-face surveys, sanitation facility spot checks, and handwashing demonstrations [20].

### 2018 survey methods

For the 2018 survey, the sample size was determined using a precision level of 2.8%, a design effect of 4.5, and an assumed prevalence of 40% for ‘handwashing location with water and soap’ (based on the 2014 baseline survey). This calculation resulted in a target sample size of 5250 households; ultimately, 5280 households were surveyed [21].

In this survey, two-stage stratified cluster sampling design was employed. In the first stage, 176 enumeration areas (EAs) were selected from a total of 293,570 EAs in Bangladesh using PPS sampling. The 2011 Population and Housing Census served as the sampling frame, with adjustments made for areas reclassified as urban. In the second stage, 30 households were systematically sampled from each selected EA. Data collection, conducted between March and May 2018, included face-to-face interviews with caregivers or heads of households, spot checks of sanitation facilities, and handwashing demonstrations involving both caregivers and children under five [21].

### Study measures

Household WASH status was assessed using the classifications and definitions provided by the WHO-UNICEF Joint Monitoring Programme (JMP). Specifically, drinking water facilities were categorized as basic, limited, unimproved, or dependent on surface water; sanitation facilities were classified as basic, limited, unimproved, or involving open defecation; and hygiene facilities were identified as basic, limited, or lacking a hygiene facility [26,27].

It is important to note that we did not apply the ‘safely managed’ classification to either drinking water or sanitation facilities. Assessing safely managed drinking water requires water quality testing, and safely managed sanitation facilities depend on the safe disposal of excreta, either through on-site containment or appropriate offsite treatment. The hygiene surveys conducted in 2014 and 2018 did not include these components [26,27].

The independent variable in this study was time, with 2014 coded as 0 and 2018 as 1. In addition, the analysis adjusted for several covariates: area of residence (urban or rural), respondent sex (male or female), mean respondent age (in years), housing ownership status (self-owned, rental, or public), household head’s sex (male or female), mean age of the household head (in years), household size, and wealth index (categorized into quintiles: poorest, second, third, fourth, and wealthiest).

### Statistical analysis

We performed descriptive statistics for primary WASH variables and demographic indicators in 2014 and 2018 across rural and urban settings. Frequencies and percentages were used for categorical variables, and means with standard deviations (SD) were computed for continuous variables. To compare differences in WASH outcomes between 2014 and 2018 across rural and urban areas, we calculated the prevalence difference (PD) along with 95% confidence intervals (CI). For binary outcome variables, we employed weighted logistic regression models, whereas linear regression was applied to continuous outcomes. The prevalence difference (PD) was derived from the average marginal effects of the regression models.

We then used binomial generalized estimating equations (GEE) models to analyze the association between household WASH outcomes and various covariates, combining data from the 2014 and 2018 surveys. To account for differences between the two survey years, we included Year as a covariate in all models (coded as 0 for 2014 and 1 for 2018).

The model is represented by the following equation:

$$\log\left(\frac{P\left(Y_{\mathrm{ij}}=1\right)}{P\left(Y_{\mathrm{ij}}=0\right)}\right)=\beta_{0}+\beta_{1}\mathrm{Yea}r_{\mathrm{ij}}+\beta_{2}\mathrm{Are}a_{\mathrm{ij}}+\cdots$$

where

$P\left(Y_{\mathrm{ij}}=1\right)$: The probability that individual i in cluster j has the WASH outcome of interest (e.g. access to a basic drinking water facility).

$P\left(Y_{\mathrm{ij}}=0\right)$: The probability that individual i in cluster j does not have the WASH outcome of interest.

$\beta_{0}$: The intercept, representing the baseline log-odds of having the WASH outcome when all predictors, is at their reference values.

$\beta_{1}$: The coefficient representing the effect of time (Year; coded as 0 for 2014 and 1 for 2018) on the log-odds of having the WASH outcome $Y_{\mathrm{ij}}=1$ compared to $Y_{\mathrm{ij}}=0$.

$\beta_{2}$: The coefficient for the effect of area (urban or rural) on the log-odds of having the WASH outcome $Y_{\mathrm{ij}}=1$ compared to $Y_{\mathrm{ij}}=0$.

Other covariates (not shown explicitly): Additional covariates (e.g. sex and age of the household head, along with other individual or household characteristics), each with an associated coefficient reflecting its effect on the log-odds of the WASH outcome.

An exchangeable correlation structure was employed to account for within-cluster correlations, ensuring robust variance estimation while considering the survey design. The model adjusted for all the covariates included in this study. This approach allows us to evaluate differences in WASH outcomes over time while accounting for individual and household characteristics.

To address cluster disparities resulting from the complex sampling design and differences in the number of clusters between urban and rural areas, we applied weighting factors in the regression models: for 2014, weights were 50/10,552 for urban areas and 50/86,925 for rural areas; for 2018, weights were 72/10,552 for urban areas and 104/86,925 for rural areas. A *p*-value below 0.05 was considered to be statistically significant throughout the analysis. Data analysis was conducted using STATA version 15.0 (Stata Corp., College Station, TX, USA).

## Results

### Differences in sociodemographic characteristics from 2014 to 2018

Table 1 presents the characteristics of rural, urban, and all households for the survey years 2014 and 2018. The ownership status of households (categorized as self-owned, rental, or public) remained stable across rural, urban, and national levels over time. The prevalence of male-headed households significantly decreased by 10.0% points in rural areas, 9.5 in urban areas, and 9.9 nationally. However, the mean age of household heads significantly increased by 7.8 years in rural areas, 4.6 years in urban areas, and 7.5 years nationally.

**Table 1.** Sociodemographic characteristics of the respondents and households.

Indicators	Rural households			Urban households			All households		
	2014 *n* (%) or mean (SD) *N* = 1250	2018 *n* (%) or mean (SD) *N* = 3120	PD^†§^ (CI)	2014 *n* (%) or mean (SD) *N* = 1250	2018 *n* (%) or mean (SD) *N* = 2160	PD^†§^ (CI)	2014 *n* (%) or mean (SD) *N* = 2500	2018 *n* (%) or mean (SD) *N* = 5280	PD^†§^ (CI)
Sex of respondents (female)	1241 (99.3)	2889 (92.6)	−9.8 (−12.6, −6.9)	1232 (98.6)	1963 (90.9)	−10.0 (−13.5, −6.4)	2473 (98.9)	4852 (91.9)	−9.7 (−12.2, −7.2)
Age of respondents (in years)	26.6 (6.7)	37.7 (13.4)	11.0 (10.2, 11.8)	27.2 (7.3)	35.5 (12.3)	8.3 (7.3, 9.3)	26.9 (7.0)	36.8 (13.0)	10.7 (10.0, 11.5)
Ownership of living house									
Self-owned	1155 (92.4)	2859 (91.6)	0 (−0.1, 0)	589 (47.1)	966 (44.7)	0 (−0.1, 0.1)	1744 (69.8)	3825 (72.4)	0 (−0.1, 0)
Rental	53 (4.2)	90 (2.9)	0 (−0.1, 0)	617 (49.4)	1005 (46.5)	0 (−0.2, 0.1)	670 (26.8)	1095 (20.7)	0 (−0.1, 0)
Public	42 (3.4)	171 (5.5)	0 (0, 0.1)	44 (3.5)	189 (8.8)	0.1 (0, 0.1)	86 (3.4)	360 (6.8)	0 (0, 0.1)
Sex of household head (male)	1222 (97.8)	2792 (89.5)	−10.0 (−12.9, −7.2)	1197 (95.8)	1884 (87.2)	−9.5 (−12.1, −6.9)	2419 (96.8)	4676 (88.6)	−9.9 (−12.4, −7.4)
Age of the household head (in years)	39.5 (13.1)	47.3 (14.0)	7.8 (6.6, 9.0)	39.1 (12.4)	43.7 (12.8)	4.6 (3.4, 5.8)	39.3 (12.8)	45.8 (13.7)	7.5 (6.4, 8.6)
Household size	5.3 (2.2)	4.5 (1.8)	−0.8 (−1.1, −0.6)	5.1 (2.2)	4.2 (1.7)	−1.0 (−1.2, −0.7)	5.2 (2.2)	4.3 (1.8)	−0.8 (−1.1, −0.6)
Socio-economic classification based on wealth index*									
Poorest	542 (43.4)	669 (21.4)	−21.0 (−27.2, −14.8)	133 (10.6)	212 (9.8)	−0.8 (−6.8, 5.1)	675 (27.0)	881 (16.7)	−18.9 (−24.8, −13.1)
2nd	275 (22.0)	809 (25.9)	3.9 (0.5, 7.4)	177 (14.2)	295 (13.7)	−0.5 (−4.9, 3.9)	452 (18.1)	1104 (20.9)	3.5 (0.3, 6.6)
3rd	169 (13.5)	714 (22.9)	9.6 (6.4, 12.8)	225 (18.0)	448 (20.7)	2.8 (−1.3, 6.8)	394 (15.8)	1162 (22.0)	8.8 (5.9, 11.7)
4th	135 (10.8)	582 (18.7)	8.1 (4.3, 11.9)	282 (22.6)	557 (25.8)	3.2 (−1.4, 7.8)	417 (16.7)	1139 (21.6)	7.5 (4.1, 11.0)
Wealthiest	129 (10.3)	346 (11.1)	0.8 (−3.0, 4.6)	433 (34.6)	648 (30.0)	−4.6 (−13.7, 4.4)	562 (22.5)	994 (18.8)	0.2 (−3.5, 3.8)
Area	1250 (50)	3120 (59.1)	0.2 (−0.2, 0.5)	1250 (50)	2160 (40.9)	0.2 (−0.2, 0.5)	2500 (100)	5280 (100)	0.2 (−0.1, 0.5)

*Used principal component analysis (PCA) method using household asset variables, respondent education, land ownership.

^†^Prevalence difference (PD) with 95% confidence interval (CI) using marginal effect.

^§^Cluster adjusted weighted logistic regression (binary variables) and linear regression (continuous variables).

Household sizes reduced in rural and urban areas, showing a PD of −0.8 in rural areas, −1.0 in urban areas, and −0.8 nationally. From 2014 to 2018, the proportion of the poorest households decreased significantly, with a PD of −21.0 in rural areas and −18.9 nationally. As a result, the second, third, and fourth wealth quintiles increased in rural areas (PDs: 3.9, 9.6, and 8.1, respectively) and nationally (PDs: 3.5, 8.8, and 7.5, respectively). Differences in socio-economic status among urban households were less pronounced and generally insignificant.

### Differences in household WASH access and status from 2014 to 2018

Table 2 summarizes changes in household WASH access and facilities between 2014 and 2018 across rural, urban, and national levels. Rural households maintained near-universal access to improved drinking water, while urban households experienced a nonsignificant decline (from 87.3% in 2014 to 86.1% in 2018). Nationally, access remained stagnant, showing no significant change. Access to basic drinking water improved non-significantly in rural areas (PD: 2.2) (Table 3). Surface water use increased slightly in urban areas, resulting in a nonsignificant national increase.

**Table 2.** Household WASH status.

Indicators	Rural households			Urban households			All households		
	2014 *n* (%) *N* = 1250	2018 *n* (%) *N* = 3120	PD^*†^ (CI)	2014 *n* (%) *N* = 1250	2018 *n* (%) *N* = 2160	PD^*†^ (CI)	2014 *n* (%) *N* = 2500	2018 *n* (%) *N* = 5280	PD^*†^ (CI)
Drinking water access									
Improved sources	1212 (97.0)	3075 (98.0)	0.01 (−0.2, 0.2)	1091 (87.3)	1859 (86.1)	−0.01 (−0.1, 0.1)	2303 (92.0)	4934 (93.5)	0.01 (−0.02, 0.04)
Collection time is not more than 30 min	1239 (99.1)	3120 (99.5)	0 (−0.01, 0.01)	1249 (99.9)	2155 (99.8)	0 (−0.01, 0)	2488 (99.5)	5259 (99.6)	0 (−0.01, 0.01)
Drinking water status according to JMP ladder									
Basic	1202 (96.2)	3069 (98.4)	2.2 (−1.5, 6.0)	1090 (87.2)	1854 (85.8)	−1 (−9, 6)	2292 (91.7)	4923 (93.2)	1.8 (−1, 5)
Limited	10 (0.8)	6 (0.19)	−0.7 (−1.6, 0.2)	1 (0.08)	5 (0.23)	0.2 (−0.3, 0.6)	11 (0.44)	11 (0.21)	−0.6 (−1.3, 0.2)
Unimproved	3 (0.2)	4 (0.1)	0 (−0.01, 0)	0 (0)	0 (0)	–	3 (0.1)	4 (0.1)	0 (−0.01, 0)
Surface water	6 (0.5)	26 (0.8)	0 (−0.01, 0.02)	0 (0)	45 (2.1)	–	6 (0.2)	71 (1.3)	0 (−0.01, 0.02)
Sanitation access									
Improved	466 (37.3)	2340 (75.0)	33.8 (28.4, 39.1)	933 (74.6)	1785 (82.6)	7.9 (−1.3, 17.1)	1399 (56.0)	4125 (78.1)	31.4 (26.2, 36.6)
Not shared facilities	719 (57.5)	2199 (70.5)	12.7 (6.8, 18.7)	701 (56.1)	1080 (50.0)	−0.06 (−0.2, 0)	1420 (56.8)	3279 (62.1)	0.11 (0.1, 0.2)
Sanitation status according to JMP ladder									
Basic	324 (25.9)	1716 (55)	27.8 (22.4, 33.2)	558 (44.6)	948 (43.9)	−0.8 (−10.4, 8.9)	882 (35.3)	2664 (50.5)	25 (19.8, 30.1)
Limited	142 (11.4)	624 (20.0)	8.9 (4.8, 12.9)	375 (30.0)	837 (38.8)	8.8 (−0.3, 17.8)	517 (20.7)	1461 (27.7)	8.8 (5.0, 12.6)
Unimproved	733 (58.6)	694 (22.2)	−32.7 (−37.9, −27.5)	313 (25.0)	368 (17.0)	−7.9 (−17, 1.2)	1046 (41.8)	1062 (20.1)	−30.5 (−35.5, −25.4)
Open defecation	51 (4.1)	86 (2.8)	−0.01 (−0.05, 0.02)	4 (0.3)	7 (0.3)	0 (−0.01, 0.01)	55 (2.2)	93 (1.8)	−0.01 (−0.04, 0.02)
Hygiene access									
Availability of a handwashing facility	687 (55.0)	2110 (67.6)	12.5 (6.2, 18.8)	1008 (80.6)	1798 (83.2)	2.6 (−4.2, 9.4)	1695 (67.8)	3908 (74.0)	11.4 (5.6, 17.2)
Availability of soap	717 (57.4)	2087 (66.9)	0.1 (0.0, 0.2)	992 (79.4)	1581 (73.2)	−0.1 (−0.1, 0.0)	1709 (68.4)	3668 (69.5)	0.1 (0.0, 0.1)
Availability of water for handwashing	940 (75.2)	2600 (83.3)	0.1 (0.0, 0.1)	1168 (93.4)	1965 (91.0)	0 (−0.1, 0.02)	2108 (84.3)	4565 (86.5)	0.1 (0.0, 0.1)
Hygiene status according to JMP ladder									
Basic	375 (30.0)	1498 (48.0)	0.2 (0.1, 0.3)	808 (64.6)	1399 (64.8)	0 (−0.1, 0.1)	1183 (47.3)	2897 (54.9)	0.2 (0.1, 0.2)
Limited	312 (25.0)	612 (19.6)	−0.1 (−0.1, −0.0)	200 (16.0)	399 (18.5)	0 (−0.0, 0.1)	512 (20.5)	1011 (19.2)	0 (−0.1, 0.0)
No facility	563 (45.0)	1010 (32.4)	−0.1 (−0.2, −0.1)	242 (19.4)	362 (16.8)	0 (−0.1, 0.0)	805 (32.2)	1372 (26.0)	−0.1 (−0.2, −0.1)

*Prevalence difference (PD) with 95% confidence interval (CI) using marginal effects.

^†^Cluster adjusted weighted logistic regression (binary variables) and linear regression (continuous variables).

**Table 3.** Effects of sociodemographic variables on WASH status.

Covariates	Coef. (95% CI)*	*p* value^†^	Coef. (95% CI)*	*p* value^†^	Coef. (95% CI)*	*p* value^†^	Coef. (95% CI)*	*p* value^†^
	**Drinking water status according to JMP ladder**							
	**Basic drinking water**		**Limited drinking water**		**Unimproved drinking water**		**Surface water**	
Difference in time (ref: year 2014)	0.7 (−0.3, 1.8)	*0.160*	−0.7 (−1.6, 0.3)	*0.172*	−2 (−4.7, 0.7)	*0.139*	0 (0, 0)	–
Sex of respondents (ref: female)	0.2 (−0.1, 0.5)	*0.191*	0 (0, 0)	–	0 (0, 0)	–	0 (0, 0)	–
Age of respondents	0 (0, 0)	*0.097*	0 (−0.1, 0.1)	*0.669*	0 (0, 0)	***0.036***	0 (0, 0)	–
Ownership of living house (ref: self-owned)								
Rental	−0.5 (−1.1, 0.2)	*0.156*	−0.6 (−2, 0.8)	*0.399*	1.9 (0.8, 3)	***0.001***	0 (0, 0)	–
Public	−0.3 (−0.8, 0.3)	*0.308*	0.8 (−0.8, 2.5)	*0.312*	0 (0, 0)	–	0 (0, 0)	–
Sex of household head (ref: female)	0.4 (−0.1, 0.8)	*0.096*	1.1 (−0.6, 2.7)	*0.205*	1 (−0.1, 2)	*0.074*	0 (0, 0)	–
Age of the household head	0 (0, 0)	*0.354*	0 (0, 0)	*0.930*	0.1 (0, 0.1)	*** <0.001***	0 (0, 0)	–
Household size	0 (−0.1, 0)	*0.408*	0.1 (0, 0.3)	*0.133*	−0.2 (−0.3, 0)	***0.011***	0 (0, 0)	–
Socio-economic status (ref: poorest)								
2nd	0.1 (−0.4, 0.5)	*0.706*	−2.7 (−4.8, −0.6)	***0.011***	−0.7 (−1.9, 0.4)	*0.192*	0 (0, 0)	–
3rd	0.1 (−0.3, 0.6)	*0.589*	−3.5 (−5.1, −1.9)	*** <0.001***	−0.7 (−1.7, 0.3)	*0.174*	0 (0, 0)	–
4th	0.2 (−0.3, 0.8)	*0.388*	−1.5 (−3.1, 0.1)	*0.061*	0 (0, 0)	–	0 (0, 0)	–
Wealthiest	0.2 (−0.5, 0.8)	*0.623*	−2.1 (−4.4, 0.3)	*0.084*	0 (0, 0)	–	0 (0, 0)	–
Area (ref: rural)	−1.4 (−2.5, −0.3)	***0.014***	−0.3 (−1.5, 0.9)	*0.601*	0 (0, 0)	–	0 (0, 0)	–
	**Sanitation status according to JMP ladder**							
	**Basic sanitation**		**Limited sanitation**		**Unimproved sanitation**		**Open defecation**	
Difference in time (ref: year 2014)	0.8 (0.5, 1)	*** <0.001***	0.7 (0.4, 1)	*** <0.001***	−1.3 (−1.7, −1)	*** <0.001***	0.8 (−0.1, 1.8)	*0.090*
Sex of respondents (ref: female)	−0.1 (−0.4, 0.2)	*0.480*	0.1 (−0.2, 0.4)	*0.499*	−0.1 (−0.4, 0.2)	*0.725*	0.2 (−0.5, 1)	*0.532*
Age of respondents	0 (0, 0)	*** <0.001***	0 (0, 0)	***0.027***	0 (0, 0)	***0.024***	0 (0, 0)	*0.850*
Ownership of living house (ref: self-owned)								
Rental	−0.9 (−1.2, −0.6)	*** <0.001***	1.2 (0.9, 1.6)	*** <0.001***	−0.7 (−1.2, −0.3)	***0.001***	−0.5 (−1.9, 0.8)	*0.422*
Public	−0.2 (−0.6, 0.2)	*0.258*	−0.1 (−0.5, 0.3)	*0.636*	0.1 (−0.2, 0.4)	*0.426*	0.2 (−0.4, 0.8)	*0.519*
Sex of household head (ref: female)	0 (−0.2, 0.2)	*0.989*	0 (−0.2, 0.3)	*0.867*	0 (−0.2, 0.2)	*0.946*	−0.4 (−0.9, 0.1)	*0.162*
Age of the household head	0 (0, 0)	***0.019***	0 (0, 0)	*** <0.001***	0 (0, 0)	***0.041***	0 (0, 0)	*0.582*
Household size	0 (0, 0)	*0.819*	−0.2 (−0.2, −0.1)	*** <0.001***	0.1 (0.1, 0.1)	*** <0.001***	0.1 (0, 0.2)	*0.091*
Socio-economic status (ref: poorest)								
2nd	1.6 (1.3, 1.8)	*** <0.001***	0.4 (0.2, 0.7)	***0.001***	−1 (−1.2, −0.8)	*** <0.001***	−2.1 (−2.7, −1.5)	*** < 0.001***
3rd	2.1 (1.8, 2.4)	*** <0.001***	0.5 (0.2, 0.8)	***0.001***	−1.6 (−1.9, −1.4)	*** <0.001***	−2.9 (−4, −1.8)	*** < 0.001***
4th	2.9 (2.6, 3.2)	*** <0.001***	0.1 (−0.3, 0.4)	*0.701*	−2.3 (−2.7, −2)	*** <0.001***	0 (0, 0)	–
Wealthiest	4.1 (3.8, 4.4)	*** <0.001***	−0.8 (−1.2, −0.3)	***0.001***	−3.6 (−4.1, −3.1)	*** <0.001***	0 (0, 0)	–
Area (ref: rural)	−0.4 (−0.7, −0.2)	***0.002***	0.6 (0.3, 1)	*** <0.001***	−0.1 (−0.5, 0.2)	*0.465*	−1.1 (−2.1, 0)	***0.050***
	**Hygiene status according to JMP ladder**							
	**Basic hygiene**		**Limited hygiene**		**No hygiene facilities**		–	
Difference in time (ref: year 2014)	0.5 (0.2, 0.7)	***0.002***	−0.3 (−0.5, −0.1)	***0.016***	−0.2 (−0.4, 0.1)	*0.125*	–	–
Sex of respondents (ref: female)	0.1 (−0.2, 0.3)	*0.734*	−0.3 (−0.6, 0)	*0.055*	0.2 (−0.1, 0.5)	*0.247*	–	–
Age of respondents	0 (0, 0)	*0.124*	0 (0, 0)	***0.019***	0 (0, 0)	*** <0.001***	–	–
Ownership of living house (ref: self-owned)								
Rental	0.4 (0.1, 0.7)	***0.003***	0 (−0.3, 0.3)	*0.884*	−0.6 (−0.9, −0.3)	*** <0.001***	–	–
Public	0.1 (−0.1, 0.4)	*0.333*	0 (−0.3, 0.3)	*0.986*	−0.1 (−0.4, 0.2)	*0.438*	–	–
Sex of household head (ref: female)	0 (−0.2, 0.3)	*0.878*	0 (−0.2, 0.2)	*0.942*	0 (−0.3, 0.2)	*0.785*	–	–
Age of the household head	0 (0, 0)	*0.704*	0 (0, 0)	*0.436*	0 (0, 0)	*0.539*	–	–
Household size	−0.1 (−0.1, 0)	***0.002***	0 (0, 0.1)	*0.482*	0.1 (0, 0.1)	***0.002***	–	–
Socio-economic status (ref: poorest)								
2nd	0.5 (0.4, 0.7)	*** <0.001***	0.2 (0, 0.4)	*0.076*	−0.6 (−0.8, −0.4)	*** <0.001***	–	–
3rd	0.9 (0.7, 1)	*** <0.001***	−0.1 (−0.3, 0.1)	*0.445*	−0.7 (−0.9, −0.5)	*** <0.001***	–	–
4th	1.5 (1.3, 1.7)	*** <0.001***	−0.6 (−0.9, −0.3)	*** <0.001***	−1.1 (−1.4, −0.9)	*** <0.001***	–	–
Wealthiest	2.4 (2.1, 2.7)	*** <0.001***	−0.9 (−1.2, −0.6)	*** <0.001***	−2.2 (−2.5, −1.9)	*** <0.001***	–	–
Area (ref: rural)	0.4 (0.1, 0.6)	***0.009***	0 (−0.3, 0.2)	*0.777*	−0.4 (−0.6, −0.1)	***0.002***	–	–

*Coefficient with 95% confidence interval (CI) estimated using binomial generalized estimating equations (GEE) models with marginal effects.

^†^Models were adjusted for clustering and survey weights using weighted.

^‡^Statistically significant results (*p* value < 0.05) are highlighted in bold.

Rural households experienced a significant increase in access to improved sanitation (PD: 33.8), while urban households showed a nonsignificant increase (PD: 7.9) (Table 2). Consequently, national access to improved sanitation increased significantly (PD: 31.4). In rural areas, access to nonshared facilities improved (PD: 12.7), whereas a slight, nonsignificant decline was observed in urban areas, resulting in no national change. Nationally, access to basic sanitation improved significantly (PD = 25), driven by rural households (PD = 27.8), with no significant change in urban areas. Limited sanitation access rose significantly in rural areas (PD = 8.9) and nationally (PD = 8.8), while urban areas showed a nonsignificant rise (PD = 8.8). Unimproved sanitation declined significantly nationwide (PD = −30.5) and in rural households (PD = −32.7), with a minor, nonsignificant reduction in urban areas (PD = −7.9). After adjusting for sociodemographic factors, households in 2018 were more likely to have basic sanitation (Coef.: 0.8; *p* < 0.001) compared to 2014 (Table 3). Access to limited sanitation also increased (Coef.: 0.7; *p* < 0.001), whereas unimproved sanitation declined (Coef.: −1.3; *p* < 0.001).

Handwashing facilities increased significantly in rural households (PD: 12.5) and at the national level (PD: 11.4), whereas urban households showed a slight, nonsignificant rise, indicating a stagnation in urban hygiene status (Table 2). Access to basic hygiene improved slightly but significantly at the national level (PD: 0.2), driven by a similar increase in rural households (PD: 0.2), while urban households remained unchanged. After adjusting for sociodemographic factors, national basic hygiene access increased (Coef.: 0.5; *p* = 0.002) and limited hygiene decreased (Coef.: −0.3; *p* = 0.016) from 2014 to 2018 (Table 3). The proportion of households without hygiene facilities decreased slightly, though this change was not statistically significant (Coef.: −0.2; *p* = 0.125).

### Effects of sociodemographic factors on drinking water status

Table 3 summarizes the impact of sociodemographic factors on WASH status. Age of the household head (Coef.: 0.1; *p* < 0.001) was positively associated with the use of unimproved drinking water, whereas larger household size was negatively associated (Coef.: −0.2; *p* = 0.011). Compared to owner-occupied households, rental housing was positively associated with unimproved drinking water access (Coef.: 1.9; *p* = 0.001). Higher socio-economic status was significantly negatively associated with access to limited drinking water, particularly in the second (Coef.: −2.7; *p* = 0.011) and third (Coef.: −3.5; *p* < 0.001) wealth quintiles. Living in urban areas was significantly associated with lower access to basic drinking water (Coef.: −1.4; *p* = 0.014).

### Effects of sociodemographic factors on sanitation status

Larger household size had a small negative effect on limited sanitation (Coef.: −0.2; *p* < 0.001) and a small positive association with unimproved sanitation (Coef.: 0.1; *p* < 0.001). Rental housing, compared to owner-occupied homes, was negatively associated with basic sanitation (Coef.: −0.9; *p* < 0.001) and unimproved sanitation (Coef.: −0.7; *p* = 0.001) but positively associated with limited sanitation (Coef.: 1.2; *p* < 0.001). Public housing showed no significant effect.

Higher socio-economic status was positively associated with basic sanitation (Coef.: 1.6–4.1; all *p* < 0.001). The second and third wealth quintiles had higher odds of limited sanitation than the poorest (Coef.: 0.4 and 0.5; *p* = 0.001), whereas the wealthiest quintile was less likely to have limited sanitation (Coef.: −0.8; *p* = 0.001). Higher socio-economic status was also negatively associated with unimproved sanitation, particularly among the wealthiest households (Coef.: −3.6; *p* < 0.001).

Urban households had lower access to basic sanitation than rural households (Coef.: −0.4; *p* = 0.002) but were more likely to have limited sanitation (Coef.: 0.6; *p* < 0.001). Urban households also showed a marginally significant lower likelihood of open defecation compared to rural households (Coef.: −1.1; *p* = 0.050).

### Effects of sociodemographic factors on hygiene status

Larger household size reduced basic hygiene access (Coef.: −0.1; *p* = 0.002) and increased the likelihood of lacking hygiene facilities (Coef.: 0.1; *p* = 0.002). Rental households had higher basic hygiene access than self-owned homes (Coef.: 0.4; *p* = 0.003) and were less likely to lack hygiene facilities (Coef.: −0.6; *p* < 0.001).

Higher socio-economic status was associated with greater basic hygiene access, with wealthier quintiles showing higher access than the poorest (Coef.: 0.5–2.4; all *p* < 0.001). Higher socio-economic status was also linked to lower access to limited hygiene, particularly in the fourth (Coef.: −0.6; *p* < 0.001) and wealthiest quintiles (Coef.: −0.9; *p* < 0.001), and with a reduced likelihood of lacking hygiene facilities (Coef.: −0.6 to −2.2; all *p* < 0.001).

Rural residence was associated with lower basic hygiene access (Coef.: −1.4; *p* = 0.014), while urban households had a significantly lower likelihood of lacking hygiene facilities (Coef.: −0.4; *p* = 0.002).

## Discussion

In this study, we examined changes in household WASH status in Bangladesh between 2014 and 2018 and the sociodemographic factors associated with access, using data from the two national hygiene surveys at national and urban = rural levels. We analyzed prevalence differences to assess temporal changes and conducted GEE analyses to evaluate the influence of sociodemographic factors. The purpose of the study was to assess changes in household WASH and identify factors influencing access to WASH facilities. Understanding these patterns can guide targeted interventions and policies to support equitable improvements in WASH services across urban and rural populations.

### Household WASH condition differences from 2014 to 2018

The study demonstrates substantial progress in household WASH, particularly in rural sanitation and hygiene facilities, while highlighting areas requiring continued attention, such as urban areas where access remains stagnant or shows signs of decline. From 2014 to 2018, rural households maintained near-universal access to improved drinking water, whereas urban households experienced a slight, nonsignificant decline, resulting in stagnant national coverage. The increasing number of city migrants in temporary settlements may have contributed to this decline [28]. Consistent with our study, recent data indicate that household access to basic water facilities in Bangladesh reached 99.5% in 2019 [11,13]. The JMP estimated that basic drinking water coverage in Bangladesh was 99% in 2024, with an annual growth rate of 0.18% points [4]. As we eventually approach universal coverage, which was the target set by the Millennium Development Goals for universal access by 2015, future progress in household drinking water access may be constrained, necessitating targeted efforts to achieve complete coverage [29].

Our study shows that between 2014 and 2018, sanitation improved substantially in rural areas, driving significant national gains in basic and improved sanitation and reductions in unimproved sanitation, while changes in urban areas were smaller. According to JMP, by 2024, 68% of the Bangladeshi population had access to at least basic sanitation services, with an average annual increase ranging from 0.65% to 1.83% points [4]. Despite this progress, the target of universal coverage of improved sanitation, initially set for 2015, remains unmet [29].

Regarding hygiene, our study found that handwashing facilities increased in rural areas and nationally, whereas urban hygiene remained largely unchanged. Overall, basic hygiene access improved slightly, limited hygiene decreased, and the proportion of households without facilities remained stable. Bangladesh achieved an annual growth of 3.34% points in basic hygiene, reaching 72% coverage by 2024 [4], consistent with our findings of significant increases in both basic and limited hygiene services between 2014 and 2018.

### Sociodemographic factors and WASH

In our study, rental housing was linked to higher basic hygiene but mixed sanitation outcomes. In Afghanistan, households living in self-owned houses are about three times more likely to have improved sanitation facilities than those in rented accommodations. This lower prevalence of improved sanitation in rental houses may be partly due to the households’ lower socioeconomic status, which limits their ability to construct such facilities [30]. However, a study in Ethiopia found no relationship between housing tenure and hand hygiene practices [31].

Our study also found that larger households in Bangladesh are less likely to use unimproved drinking water. However, they also showed slightly higher use of limited and unimproved sanitation and were marginally more likely to lack hygiene facilities. Recent studies in Bangladesh reported that households with greater members are more likely to have basic WASH facilities [9,13]. Conflicting patterns have been observed in coastal Bangladesh, where larger households were less likely to have basic sanitation [32], and in urban slums in eastern India, where household overcrowding was associated with poorer WASH practices [33]. Evidences from Afghanistan and Ghana suggest that smaller households generally have better access to WASH facilities [9,34]. While larger households may increase latrine ownership in some contexts, resource constraints can limit construction, and outcomes are further influenced by household wealth and sanitation quality [35], highlighting the context-dependent effects of household size on WASH access.

Socioeconomic status emerged as a strong determinant of WASH access. In 2019 in Bangladesh, individuals in the wealthiest quintile were 4.4 times more likely to access basic WASH services compared to the poorest quintile, with the likelihood varying by service type: 1.1 times for drinking water, 1.7 times for sanitation, and 2.8 times for hygiene [4,11]. Similarly, our analysis found a strong positive association between higher socioeconomic status and improved WASH outcomes across nearly all categories. These results are consistent with recent studies in Bangladesh [13,32] and corroborate findings from South and Southeast Asia as well as Africa [8,9,34,36–41], where wealth remains a key determinant of access to improved water and sanitation. The inability of poorer households to afford these services contributes to persistent inequalities [39]. These results further support the notion that GDP per capita is a crucial indicator for achieving water security [40].

Our findings also revealed notable urban–rural disparities. Urban households in Bangladesh had lower access to basic drinking water and sanitation but higher access to basic hygiene facilities compared to rural households. In 2024, basic drinking water coverage was nearly universal in both urban and rural areas, while basic sanitation coverage was similar at 68% in each. However, basic hygiene coverage was higher in urban areas (80%) compared to rural areas (66%) [4]. Previous studies in Bangladesh have shown that, after accounting for household and demographic factors, rural areas were 1.6 times more likely to have all three basic WASH services compared to urban areas [13]. This disparity may be explained by the concentration of densely populated informal settlements in cities, where WASH infrastructure is limited [42].

Across South Asia, urban households generally have better access to WASH services compared to rural households, highlighting the benefits of urban infrastructure and the challenges faced in rural areas [9]. Similarly, studies from other developing countries indicate that rural households often experience poorer access to safe drinking water and hygiene services, with socioeconomic development serving as a key determinant of sanitation access [10,37,38,43,44]. Addressing these persistent inequalities will require sustainable and inclusive strategies, along with coordinated government and private sector efforts, to improve equitable WASH access and strengthen public health [16].

### Recommendations and implication of the study

The findings of this study have important implications for WASH policy and practice in Bangladesh. Urban areas, where access to basic drinking water and sanitation remained stagnant or declined, require targeted interventions to enhance water supply, sanitation infrastructure, and hygiene facilities, particularly in densely populated informal settlements. Rural areas made substantial gains in sanitation and handwashing facilities, which should be sustained and expanded, with an emphasis on improving access to nonshared services. Socio-economic disparities were evident, as smaller and rental households were less likely to have improved WASH facilities, underscoring the need for policies that prioritize vulnerable populations. Overall, this study provides a comprehensive, evidence-based assessment of changes in WASH access between 2014 and 2018, using a robust methodology with large, nationally representative samples and advanced statistical analyses. It documents progress, highlights persistent urban–rural and socio-economic disparities, and offers actionable insights for designing equitable and effective interventions.

## Conclusion

This study provides a comprehensive assessment of household WASH status in Bangladesh between 2014 and 2018, analyzing temporal changes and sociodemographic determinants at both the national level and across urban and rural settings. The findings reveal persistent urban–rural disparities and identify socio-economic status, household size, and housing tenure as key predictors of WASH access. Rural areas achieved notable improvements in sanitation and hygiene, while urban progress remained limited, reflecting the challenges posed by rapid urbanization and the expansion of informal settlements.

From a policy perspective, this study provides practical insights for improving WASH service delivery. Urban areas require targeted investments to strengthen sanitation and hygiene facilities, particularly in densely populated informal settlements. Rural progress should be sustained through continued support for nonshared sanitation and hygiene services. Policies must prioritize vulnerable populations, especially low-income and rental households, to reduce inequalities and ensure that public investment in infrastructure and behavior change communication remains equitable and effective.

The implications of this research extend beyond Bangladesh. Many countries in South and Southeast Asia face comparable challenges, including uneven progress, rapid urbanization, and persistent socio-economic disparities in WASH access. Bangladesh’s experience, particularly its advancements in rural sanitation and hygiene, offers valuable lessons for regional and global initiatives aiming to achieve Sustainable Development Goal 6 on clean water and sanitation. Policymakers and development partners in similar contexts can apply these insights to design evidence-based, context-specific strategies that promote equitable and sustainable improvements in WASH services.

While the study provides valuable insights, it is subject to several limitations that should be considered when interpreting the findings. The absence of ‘safely managed’ classifications for drinking water and sanitation limited our ability to provide a comprehensive assessment of WASH status. We excluded notable variables such as education and employment due to the lack of directly comparable questions across the two surveys and differences in primary respondent types. The 2014 survey primarily involved caregivers of children under five, while the 2018 survey included both caregivers and household heads, making direct comparison or integration of some variables in the merged dataset unsuitable. Additionally, the cross-sectional design restricts the capacity to establish causal relationships, while the reliance on self-reported data may introduce potential biases, particularly regarding sensitive sanitation topics. Future research should consider longitudinal designs, include a wider range of sociodemographic and behavioral variables, and evaluate the long-term outcomes of targeted WASH interventions in both rural and urban areas.

## Supplementary Material

STROBE_Checklist_Cross_Sectional_Filled_Inserted_Final_8 Nov_2025.docx

## Data Availability

The data that support the findings of this study are available from the corresponding author upon reasonable request. Due to privacy and ethical restrictions, some data may not be publicly available.
