# Supplementary material for: Great strides, yet a long way to go: a comparative analysis of WASH conditions and associated sociodemographic factors from national hygiene surveys, 2014 and 2018
Source: Glob Health Action. 2026 Feb 2;19(1):2611646. doi: 10.1080/16549716.2025.2611646 (PMC12865832; doi:10.1080/16549716.2025.2611646)
Supplement: STROBE_Checklist_Cross_Sectional_Filled_Inserted_Final_8 Nov_2025.docx [file ZGHA_A_2611646_SM6435.docx]

STROBE Statement—Checklist of items that should be included in reports of ***cross-sectional studies***

|  | Item No | Recommendation |
| --- | --- | --- |
| **Title and abstract** | 1 | (*a*) Study’s design (repeated cross-sectional study) is indicated in the methods section of the abstract |
|  |  | (*b*) An informative and balanced summary of what was done and what was found is provide in the abstract |
| Introduction | | |
| Background/rationale | 2 | Scientific background (Pages 3-5) and rationale for the investigation is reported in the background section (Line 98-100) |
| Objectives | 3 | Specific objectives are satiated in the background section (Lines 102-105) |
| Methods | | |
| Study design | 4 | Key elements of study design are explained in the first paragraph of the methods section- data sources sub-section (Page 5 and Lines 110-112) |
| Setting | 5 | The setting, locations, and relevant dates, including periods of recruitment, and data collection are described in “2014 baseline survey methods” and 2018 “survey methods” sub-sections of the “Methods” section (Pages 5-6) |
| Participants | 6 | (*a*) The eligibility criteria, and the sources and methods of selection of participants are described in “2014 baseline survey methods” and “2018 survey methods” sub-sections of the “Methods” section (Pages 5-6) |
| Variables | 7 | Study variables are defined in the “Study measures” sub-section in the “Methods” section (Pages 7-8) |
| Data sources/ measurement | 8* | For each variable of interest, sources of data and details of methods of assessment (measurement) are described in “2014 baseline survey methods” and “2018 survey methods” and “Study measures” sub-sections of the “Methods” section (Pages 7-8) |
| Bias | 9 | Potential sources of bias are described in the “Conclusion” section (Page 26, Line 408-419) |
| Study size | 10 | Sample size is discussed in the “Data sources” sub-section of the “Methods” section (Lines 111-119) |
| Quantitative variables | 11 | Explanation about how quantitative variables were handled in the analyses and which groupings were chosen and why are provided in the “Study measures” sub-section in the “Methods” section (Pages 7-8) |
| Statistical methods | 12 | (*a*) Description of all statistical methods, including those used to control for confounding are provided in the “Statistical analysis” sub-section of the “Methods” section (Pages 8-9) |
|  |  | (*b*) Methods used to examine subgroups and interactions are provided in the “Statistical analysis” sub-section of the “Methods” section (Pages 8-9) |
|  |  | (*c*) Missing data were not explicitly addressed in this study because there were no missing data in both the datasets merged for this study |
|  |  | (*d*) Analytical methods taking account of sampling strategy (weights, cluster design) are described in the “Statistical analysis” sub-section of the “Methods” section (Lines 201-206) |
|  |  | (*e*) Not applicable; sensitivity analysis was not conducted in this study. |
| Results | | |
| Participants | 13* | (a) Numbers of individuals at each stage of study are discussed in the “Data sources” sub-section of the “Methods” section (Lines 116-118) |
|  |  | (b) There was no non-participation (secondary data analysis; participation handled by original surveys) |
|  |  | (c) Flow diagram of participants recruitment is showed in Figure 1: Dataset merging flow diagram (Page 7) |
| Descriptive data | 14* | (a) Characteristics of study participants are described in “Differences in socio-demographic characteristics from 2014 to 2018” sub-section of the “Results” section (Page 10, Lines 209-220, Table 1) |
|  |  | (b) Missing data were not explicitly reported in this study |
| Outcome data | 15* | Number of summary measures are reported in the “Differences in household WASH access and status from 2014 to 2018” sub section of the “Results” section (Page 13, Lines 229-254, Table 2) |
| Main results | 16 | (*a*) Unadjusted estimates and confounder-adjusted estimates and their precision are described in the “Effects of socio-demographic factors on drinking water status”, “Effects of socio-demographic factors on sanitation status” and “Effects of socio-demographic factors on hygiene status” sub sections of the “Results” section (Page 17-21, Lines 258-297, Table 3) |
|  |  | (*b*) Continuous variables categorization is described in the “Study measures” sub-section of the “Methods” section (Line 167) |
|  |  | (*c*) Estimation of relative risk into absolute risk is not applicable for this study |
| Other analyses | 17 | Subgroup analysis by socio-demographic factors is reported in the “Effects of socio-demographic factors on drinking water status”, “Effects of socio-demographic factors on sanitation status” and “Effects of socio-demographic factors on hygiene status” sub sections of the “Results” section (Lines 258-297, Table 3) |
| Discussion | | |
| Key results | 18 | Key findings are summarized and discussed in reference to study objectives in the “Discussion” section (Lines 299-305) |
| Limitations | 19 | Limitations of the study are discussed in the “Conclusion” section (Line 408-417) |
| Interpretation | 20 | Overall interpretation of results considering objectives, limitations,  multiplicity of analyses, results from similar studies, and other relevant evidence are discussed in the “Discussion” and “Conclusion” sections (Lines 298-419) |
| Generalisability | 21 | Generalisability (external validity) of the study results are discussed in the “Conclusion” sections (Lines 401-407) |
| Other information | | |
| Funding | 22 | This study did not receive any specific funding from public, commercial, or non-profit organizations (Line 452-453) |

*Give information separately for exposed and unexposed groups.

**Note:** An Explanation and Elaboration article discusses each checklist item and gives methodological background and published examples of transparent reporting. The STROBE checklist is best used in conjunction with this article (freely available on the Web sites of PLoS Medicine at http://www.plosmedicine.org/, Annals of Internal Medicine at http://www.annals.org/, and Epidemiology at http://www.epidem.com/). Information on the STROBE Initiative is available at [www.strobe-statement.org](http://www.strobe-statement.org).

## Revised STROBE Checklist for Cross-Sectional Studies (Based on Final Revised Manuscript)

| Item No. | Recommendation | Reported on Page/Section |
| --- | --- | --- |
| 1a | Indicate the study’s design with a commonly used term in the title or abstract. | Title, Abstract |
| 1b | Provide in the abstract an informative and balanced summary of what was done and what was found. | Abstract |
| 2 | Explain the scientific background and rationale for the investigation being reported. | Introduction (Background section, Pages 3-5) |
| 3 | State specific objectives, including any prespecified hypotheses. | Abstract, Introduction (Objective paragraph, Page 5) |
| 4 | Present key elements of study design early in the paper. | Methods: Data Sources and Survey Methods (Pages 5-7) |
| 5 | Describe the setting, locations, and relevant dates, including periods of recruitment, exposure, follow-up, and data collection. | Methods: Data Sources and Survey Methods (Pages 5-7) |
| 6a | Give the eligibility criteria, and the sources and methods of selection of participants. | Methods: Data Sources and Survey Methods (Pages 5-7) |
| 7 | Clearly define all outcomes, exposures, predictors, potential confounders, and effect modifiers. | Methods: Study Measures (Pages 9-10) |
| 8 | For each variable of interest, give sources of data and details of methods of assessment (measurement). | Methods: Study Measures (Pages 7-8) |
| 9 | Describe any efforts to address potential sources of bias. | Methods (Page 9, Lines 201-204) Conclusion (Page 26, Lines 408-417) |
| 10 | Explain how the study size was arrived at. | Methods: Data Sources and Survey Methods (Pages 5-7) |
| 11 | Explain how quantitative variables were handled in the analyses. | Methods: Statistical Analysis (Pages 10-11) |
| 12a | Describe all statistical methods, including those used to control for confounding. | Methods: Statistical Analysis (Pages 7-9) |
| 12b | Describe any methods used to examine subgroups and interactions. | Methods: Statistical Analysis (Pages 7-9) |
| 12c | Explain how missing data were addressed. | Missing data were not explicitly addressed in this study because there were no missing data in both the datasets merged for this study |
| 12d | Describe analytical methods taking account of sampling strategy. | Methods: Statistical Analysis (Pages 8-9) |
| 12e | Describe any sensitivity analyses. | Not applicable; sensitivity analysis was not conducted in this study. |
| 13a | Report numbers of individuals at each stage of study. | Methods: Data Sources and Figure 1 (Page 5-7) |
| 13b | Give reasons for non-participation at each stage. | Not applicable (secondary data analysis; participant selection by original surveys) |
| 13c | Consider use of a flow diagram. | Figure 1: Dataset merging flow diagram (Page 7) |
| 14a | Give characteristics of study participants and information on exposures and potential confounders. | Results: Table 1 (Pages 10-12) |
| 14b | Indicate number of participants with missing data for each variable of interest. | Missing data were not explicitly addressed in this study because there were no missing data in both the datasets merged for this study |
| 15 | Report numbers of outcome events or summary measures. | Results: Tables 2 and 3 (Pages 13-21) |
| 16a | Give unadjusted and, if applicable, confounder-adjusted estimates and their precision. | Results: Table 3 (Pages 17-21), Statistical analysis (Pages 8-9) |
| 16b | Report category boundaries when continuous variables were categorized. | Methods: Study Measures (Line 167) |
| 16c | If relevant, consider translating estimates of relative risk into absolute risk for a meaningful time period. | Not applicable (prevalence difference and coefficients used). |
| 17 | Report other analyses done—e.g., subgroup analyses, interactions, sensitivity analyses. | Results: Subgroup analysis by socio-demographic factors (Table 3, Pages 17-21) |
| 18 | Summarize key results with reference to study objectives. | Discussion: Key Results (Pages 22) |
| 19 | Discuss limitations of the study, including sources of potential bias or imprecision. | Conclusion (Lines 408-417) |
| 20 | Give a cautious overall interpretation of results. | Discussion and Conclusion (Pages 22-26) |
| 21 | Discuss the generalisability of the study results. | Conclusion (Pages 401-407) |
| 22 | Give the source of funding and the role of funders. | Funding Information (Page 28) |
